# Structural characterization of a protective epitope spanning A(H1N1)pdm09 influenza virus neuraminidase monomers

**DOI:** 10.1038/ncomms7114

**Published:** 2015-02-10

**Authors:** Hongquan Wan, Hua Yang, David A. Shore, Rebecca J. Garten, Laura Couzens, Jin Gao, Lianlian Jiang, Paul J. Carney, Julie Villanueva, James Stevens, Maryna C. Eichelberger

**Affiliations:** 1 Division of Viral Products, Center for Biologics Evaluation and Research, Food and Drug Administration, 10903 New Hampshire Avenue, Silver Spring, Maryland 20993, USA; 2 Influenza Division, National Center for Immunization and Respiratory Diseases, Centers for Disease Control and Prevention, 1600 Clifton Road, Atlanta, Georgia 30333, USA

## Abstract

A(H1N1)pdm09 influenza A viruses predominated in the 2013–2014 USA influenza season, and although most of these viruses remain sensitive to Food and Drug Administration-approved neuraminidase (NA) inhibitors, alternative therapies are needed. Here we show that monoclonal antibody CD6, selected for binding to the NA of the prototypic A(H1N1)pdm09 virus, A/California/07/2009, protects mice against lethal virus challenge. The crystal structure of NA in complex with CD6 Fab reveals a unique epitope, where the heavy-chain complementarity determining regions (HCDRs) 1 and 2 bind one NA monomer, the light-chain CDR2 binds the neighbouring monomer, whereas HCDR3 interacts with both monomers. This 30-amino-acid epitope spans the lateral face of an NA dimer and is conserved among circulating A(H1N1)pdm09 viruses. These results suggest that the large, lateral CD6 epitope may be an effective target of antibodies selected for development as therapeutic agents against circulating H1N1 influenza viruses.

## Abstract (web summary)

Neuraminidase inhibitors offer a line of defence against flu infections, but resistance can occur even in the absence of prior exposure. Here Wan *et al*. describe the mode of action of CD6, a monoclonal antibody that protects against a common influenza strain, as a new therapeutic intervention model.

## Graphical abstract

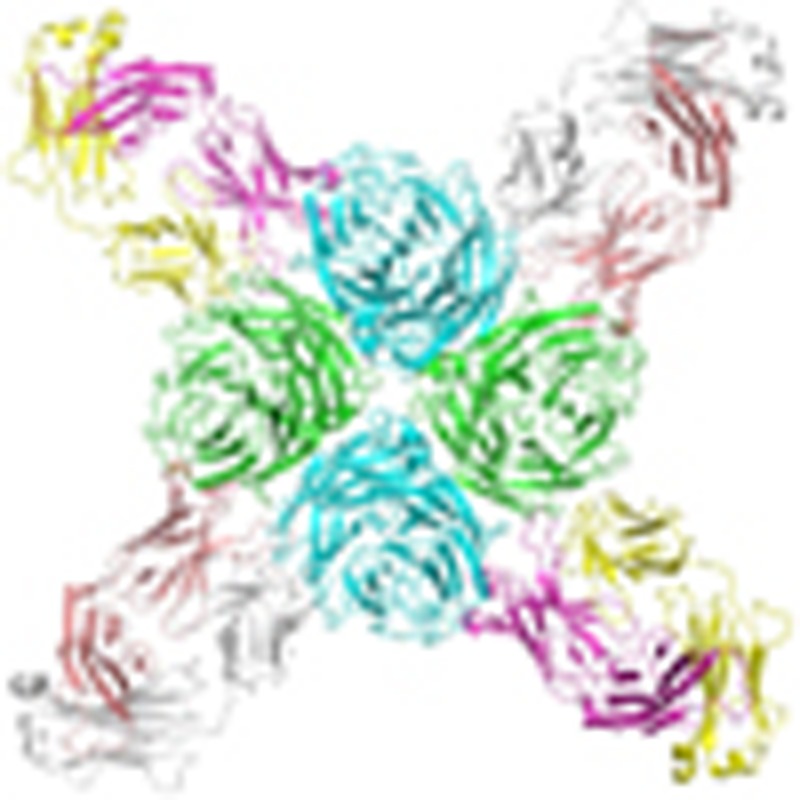

In 2009, a novel swine-origin H1N1 virus emerged in humans, resulting in the first influenza pandemic of the twenty-first century. This A(H1N1)pdm09 (pH1N1) virus has continued to circulate and was the predominant strain in the United States of America during the most recent 2013–2014 influenza season^1^. Although the overall disease incidence was lower in the 2013–2014 winter than during the 2009 outbreak, adults 18–64 years old were at higher risk of severe disease and death when compared with the traditionally highest risk 65+ year age group. This corresponded to a low rate of vaccination in young adults^2^. Fortunately, most pH1N1 viruses are sensitive to licensed neuraminidase (NA) inhibitors oseltamivir and zanamivir, and therefore patients can be offered treatments early after infection^3^. Oseltamivir is often the treatment of choice because it is available as an oral formulation and therefore easier to administer than inhalation of zanamivir. However, NA inhibitor-resistant influenza viruses can be selected quickly in treated patients^4^, or can sometimes emerge without an apparent link to treatment^5^. Indeed, during the 2013–2014 influenza season, oseltamivir-resistant pH1N1 viruses were reported in China, Japan and the United States of America^6^. The increased pH1N1 influenza activity and the emergence of oseltamivir-resistant pH1N1 viruses add urgency to the need for additional influenza antivirals. It would be advantageous for the new therapeutics to inhibit influenza virus through mechanisms distinct from oseltamivir and zanamivir, so that NA inhibitor-resistant viruses remain sensitive to the new drugs.

Medicinal compounds that are under development to fill this need include agents that target influenza’s replication machinery (for example, favipiravir), destroy the host receptors (for example, Fludase)^7^ or mask host receptors^8^. Intravenous immunoglobulin^9^,^10^,^11^ and influenza-specific monoclonal antibodies (mAbs), particularly those that bind to the conserved, stem region of the haemagglutinin (HA)^12^,^13^,^14^, are also being considered as alternative treatments. mAbs that inhibit the enzyme activity of NA also have the potential to serve as therapeutic agents^15^,^16^,^17^,^18^. NA-inhibiting antibodies are expected to have similar effectiveness against influenza as chemical NA inhibitors. However, as the mechanisms underlying enzyme inhibition are different (oseltamivir and zanamivir bind within the NA-active pocket and interrupt the enzyme reaction, whereas antibodies usually bind to epitopes surrounding the active pocket and inhibit NA activity by restricting access of the natural, large glycoconjugate substrates to the active site), the efficacy of NA-specific antibodies is unlikely to be impacted by changes in sequence that result in oseltamivir or zanamivir resistance. Consequently, NA-specific mAbs, especially those that bind to conserved epitopes, are ideal therapeutic candidates against seasonal and pandemic influenza.

Even though H1N1 and H5N1 viruses are significant threats to public health, antigenic epitopes of subtype N1 are not well characterized. We recently mapped the antigenic sites of the NA of a seasonal H1N1 virus, A/Brisbane/59/2007 (BR/07) and identified a site that is conserved in the NA of seasonal H1N1, pH1N1 and H5N1 viruses. Prophylactic treatment with mAbs specific for this antigenic site protected mice against lethal challenge with the homologous and heterologous N1-containing viruses, including pH1N1. However, these antibodies were less effective in inhibiting pH1N1 virus than the homologous virus^15^. We subsequently generated mAbs against the NA of pH1N1 virus A/California/07/2009 (CA/09). Here we report the characterization of one of these mAbs, CD6, which is effective in inhibiting pH1N1 virus in both *in vitro* and *in vivo* studies, with a focus on the crystallographic analysis of the CA/09 NA in complex with CD6 Fab. Our study reveals a unique epitope bridging neighbouring NA monomers, with a large number of contacts between antibody and antigen. Importantly, this epitope is conserved among circulating pH1N1 viruses, representing an attractive target for the development of novel therapeutics against influenza.

## Results

### Functional characteristics of pH1N1 NA-specific mAb CD6

**Figure 1:**
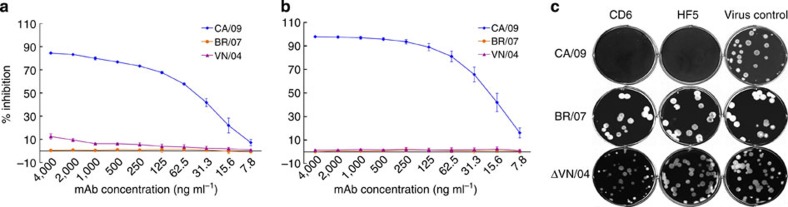
mAb CD6 inhibits pH1N1 virus NA activity. Inhibition of N1 enzyme activity by (**a**) CD6 and (**b**) HF5 measured in ELLA. The assay used fetuin as substrate and H6 reassortant viruses with the NA of CA/09 (pH1N1), BR/07 (seasonal H1N1) and VN/04 (H5N1) as the antigen/enzyme. Data are shown as mean±s.d. of three independent experiments. (**c**) Reduction of influenza virus plaque size by CD6 and HF5. MDCK cells were inoculated with CA/09, BR/07 or ΔVN/04 viruses and incubated for 3 days with an agar overlay containing no mAb (virus control), or 1 μg ml^−1^ of each mAb. Cells were stained with crystal violet to visualize the plaques.

Hybridomas secreting mAbs CD6 and HF5 were established through a fusion of Sp2/0 myeloma cells and splenocytes from mice immunized with CA/09. Both mAbs are of IgG2a isotype. The NA specificity of the mAbs was confirmed with cell-based ELISA, using 293T cells transiently expressing the NA of CA/09 as antigen^15^. In ELISA using virus-coated plates, both mAbs bound to CA/09, but did not bind a seasonal H1N1 virus BR/07, or the attenuated H5N1 virus A/Vietnam/1203/2004 (ΔVN/04) (data not shown). The affinity (Kd) of CD6 to recombinant CA/09 NA, measured with a BioLayer Interferometry Octet Red system (Fortebio Inc.), was ≈95 nM, however, binding of HF5 to recombinant NA was not measurable in this kinetic binding analysis. Both CD6 and HF5 antibodies blocked NA activity in an enzyme-linked lectin assay (ELLA), resulting in decreased cleavage of fetuin by CA/09 NA (Fig. 1a,b), with median inhibition concentrations (IC_50_) of 61±4 and 21±5 ng ml^−1^, respectively. In plaque assays with Madin–Darby canine kidney (MDCK) cells, both mAbs significantly reduced CA/09 plaque size (Fig. 1c). Although the two antibodies were specific for CA/09 NA and did not reduce enzyme activity or plaque size of BR/07, CD6 inhibited the NA activity of VN/04 to a low but measurable degree at high concentrations in ELLA (Fig. 1a). Similarly, there was a slight reduction in the size of plaques formed by ΔVN/04 virus in the presence of CD6, but not HF5 (Fig. 1c).

### mAb CD6 is an effective treatment against influenza in mice

**Figure 2:**
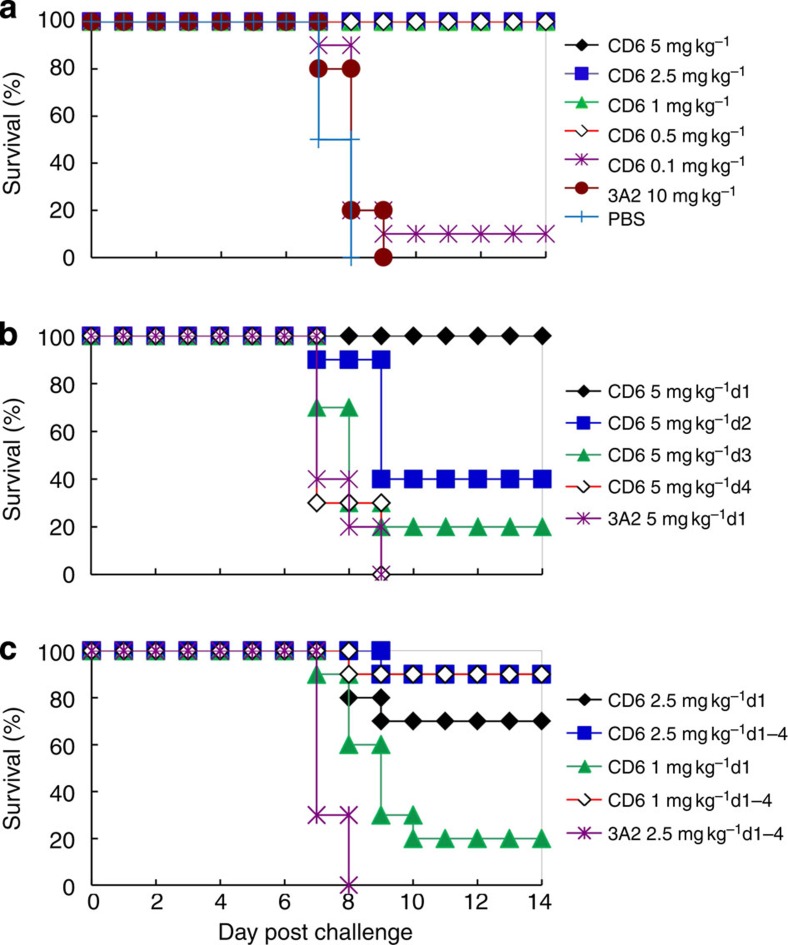
mAb CD6 protects DBA/2 mice against lethal pH1N1 virus challenge. Survival of groups of DBA/2 mice (*n*=10) treated i.p. with mAb CD6 (**a**) prophylactically at 12 h before virus challenge, (**b**) therapeutically with a single 5 mg kg^−1^ dose on different days after virus challenge or (**c**) therapeutically with either a single dose or sequential doses after virus challenge. mAb 3A2 that is specific to the seasonal H1N1 BR/07 virus was used as a negative control. In all experiments, mice were infected intranasally with 10 LD_50_ of CA/09-X179A.

As mAb HF5 failed to bind recombinant NA, we focused on the characterization of CD6, and HF5 was included in some assays as control. To evaluate the potential of mAb CD6 to protect against influenza, we monitored weight loss and survival of DBA/2 mice infected with 10 median lethal doses (LD_50_) of CA/09-X179A either before or after CD6 treatment. In the prophylactic experiments, CD6 was administered intraperitoneally (i.p.) to groups of 15 mice 12 h before virus challenge. Mouse mAb 3A2 that is specific for the NA of seasonal H1N1 virus BR/07 (ref. 15) was used as control. A single dose of CD6 at ≥0.5 mg kg^−1^ protected all mice from death (Fig. 2a) and resulted in <12% weight loss (Supplementary Fig. 1a). In contrast, the control mAb did not protect against weight loss or death. As expected, CD6 did not prevent infection, but resulted in significant reduction of lung virus titres that was proportional to the dose of mAb administered (Supplementary Fig. 1b).

CD6 was also an effective therapeutic treatment for mice infected with CA/09-X179A (10 LD_50_). A single dose of 5 mg kg^−1^ given on day 1 post challenge (p.c.) protected all mice against death and weight loss (Fig. 2b and Supplementary Fig. 1c). Protection was dependent on the time of mAb treatment, with administration of the same dose on either day 2 or day 3 p.c. resulting in survival of 40% and 20% of mice, respectively. None of the mice survived when the antibody was administered 4 days after infection. The therapeutic effectiveness of CD6 was also dependent on dose; whereas treatment with 5 mg kg^−1^ on day 1 p.c. protected all mice against death, only 70% and 20% of mice treated with 2.5 and 1 mg kg^−1^, respectively, survived. Survival at these low antibody doses was increased by repeat administration of mAb; 90% of mice survived when treated daily (on days 1, 2, 3 and 4 p.c.) with 2.5 or 1 mg kg^−1^ CD6 (Fig. 2c). The daily treatment with low CD6 doses only had a small impact on weight loss (Supplementary Fig. 1d).

### The CD6 epitope is at the interface of adjacent NA monomers

Structural analysis provides the only means to fully elucidate an antibody epitope and to identify the interactions that contribute to antibody binding. We therefore determined the crystal structure of the CD6 Fab and CA/09 NA (CD6 Fab/NA) complex at 2.8 Å resolution. The purification, crystallization and refinement of CD6 Fab/NA complex are described in the Methods, and statistics for data processing and refinement are presented in Table 1.

Comparison with NA structures previously reported^19^,^20^,^21^ suggests that the binding of CD6 does not induce any major conformational changes to the CA/09 NA structure. Although the 150-loop of the active site of pH1N1 NA has been reported in both an open and closed conformation^19^,^20^, in this Fab/NA complex structure, the CA/09 NA 150-loop adopts the reported open conformation. Additional positive electron density was observed at three of the four potential *N*-linked glycosylation sites Asn88, Asn146 and Asn235. Nine sugar residues were manually fitted into the density at these positions. These glycosylated sites are not within the CD6 epitope.

**Figure 3:**
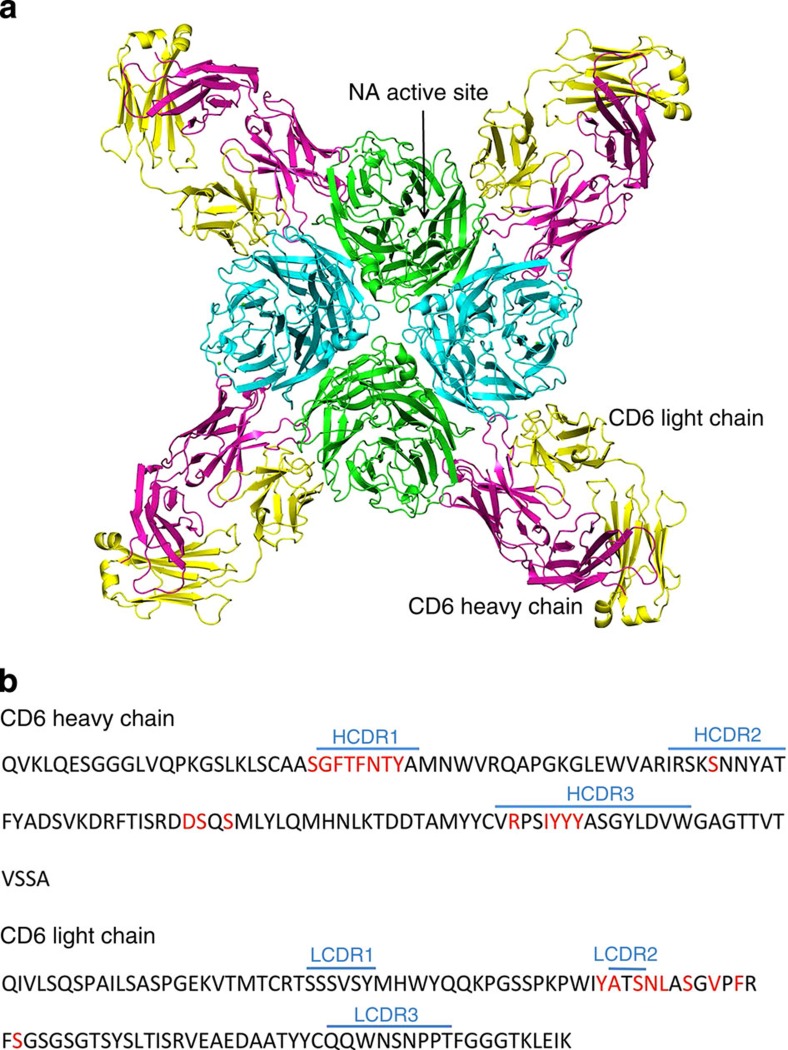
Crystal structure of CD6 Fab in complex with CA/09 NA. (**a**) Overall structure of the antibody–antigen complex. Four Fabs bind to the NA tetramer, and each Fab (H chain in magenta, L chain in yellow) bridges neighbouring NA monomers. (**b**) Amino-acid sequences of the CD6 Fab variable regions. Residues that are in contact with NA in the complex structure are highlighted in red. The CDR regions were defined using IMGT/V-QUEST^53^.

**Figure 4:**
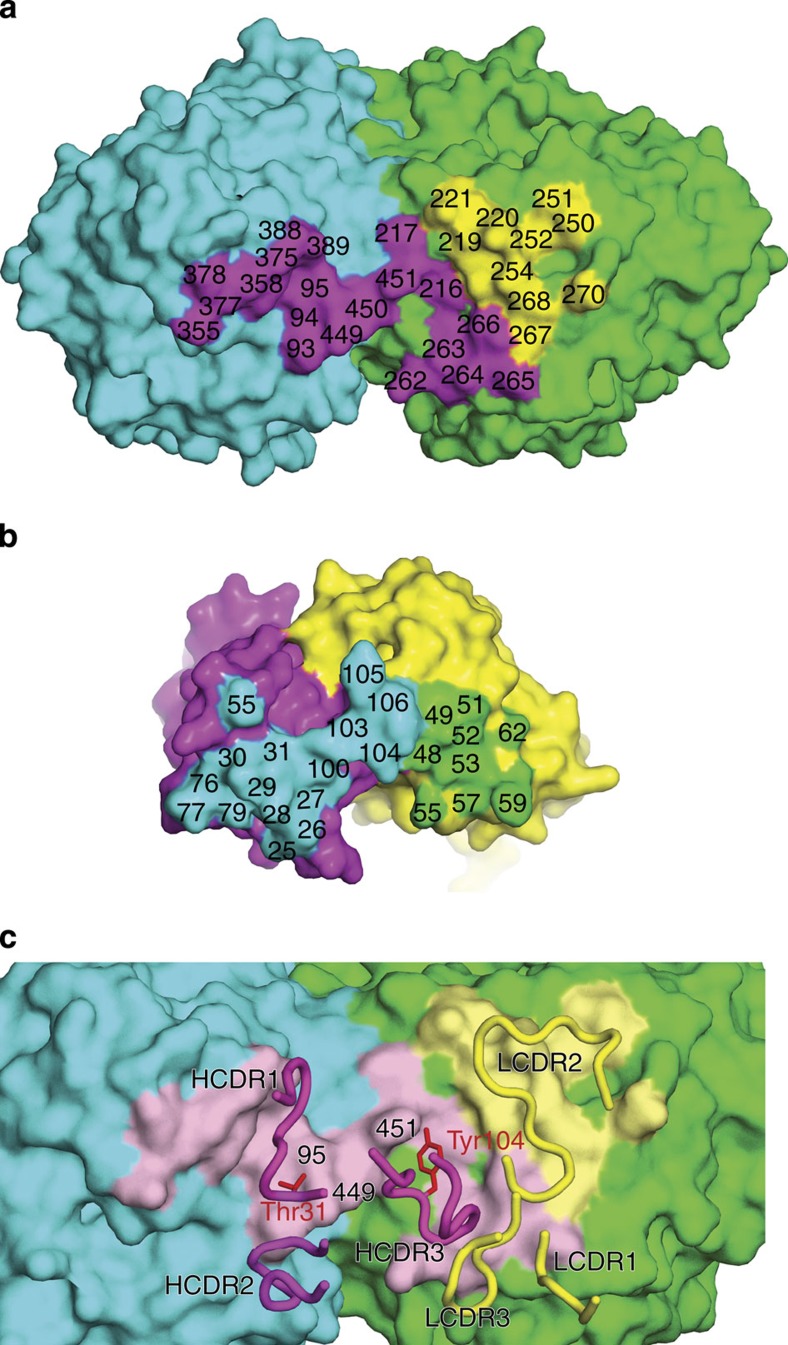
Antibody–antigen recognition by CD6. (**a**) Footprint of CD6 on NA. Two NA monomers are coloured cyan and green, respectively. NA residues interacting with the H chain are coloured in magenta and the L chain in yellow. The interacting residues of NA are labelled. (**b**) Footprint of NA on CD6 (colour theme is the same as in **a**). The interacting residues of CD6 are labelled. (**c**) Position of the CD6 CDRs on CA/09 NA. Residues interacting with the H chain are coloured in pink and the L chain in pale yellow. CDR loops are shown as tubes. Ser95, Asn449 and Asp451 comprise a focal point of interactions with the H chain of CD6, forming hydrogen bonds to Thr31 and Tyr104 on the HCDR1 and HCDR3, respectively (shown as red sticks).

To date, three epitopes on NA have been structurally characterized, two from N9 NA-Ab complexes^22^,^23^ and one from an N2 NA–Ab complex^24^. Similar to these complexes, four CD6 Fabs bind to one NA tetramer. However, the CD6 epitope is novel, forming an antibody footprint that bridges two adjacent monomers in the NA tetramer (Fig. 3a). The CD6 heavy (H) and light (L) chains both contribute to the antibody footprint (Fig. 3b). Altogether there are 30 amino acids within NA that are in contact with the antibody. The H chain of CD6 Fab makes the majority of the antigen–antibody contacts, and forms a bridge between the neighbouring NA monomers. The area on each monomer that is buried by CD6 was calculated by Proteins, Interfaces, Structures and Assemblies (PISA)^25^ as 187 and 528 Å^2^ (Fig. 4a). A large surface area of H and L chains (497 and 392 Å^2^) is buried (Fig. 4b) and, as for other NA–Fab complexes, the interacting faces of antigen and antibody show excellent shape complementarity.

The residues on the HCDR1 loop form multiple interactions with residues of one NA monomer: Pro93, Val94, Ser95, Trp358, Trp375, Ser388, Ile389, Asn449, Ser450 and Asp451 (Supplementary Table 1 and Supplementary Fig. 2a). Residues Asn355, Pro377 and Asn378 of the same monomer have interactions with residues close to the HCDR2 loop, whereas Asn449 and Ser450 on this same monomer have hydrophobic interactions with Ile103 in the HCDR3 loop. Asp451 of the NA interacts with the HCDR3 loop, forming both a hydrogen bond with Tyr104 and an indirect interaction with Arg100 through a water molecule. Ser95, Asn449 and Asp451 are in close proximity to one another, forming a focal point of interactions with HCDR1 (including a hydrogen bond to Thr31) and HCDR3 (including a hydrogen bond to Tyr104; Fig. 4c). HCDR3 is unique in that this loop extends between the NA monomers. Ile103, Tyr104, Tyr105 and Tyr106 at the tip of the HCDR3 loop consequently are in positions to have hydrophobic interactions with the main chain atoms of residues in both NA monomers: Asn449, Ser450, Asp451 of one monomer and Ile216, Lys262, Ile263, Val264, Lys265 and Ser266 of the neighbouring monomer (Supplementary Fig. 2a). For the L chain, only the residues in and around the LCDR2 loop contribute to the antibody footprint, forming hydrophobic interactions with the second NA monomer residues Trp219, Arg220, Gln250, Ala251, Ser252, Lys254, Ser266, Val267, Glu268 and Asn270 (Supplementary Table 1 and Supplementary Fig. 2b).

### Identification of amino acids critical for CD6 binding

**Figure 5:**
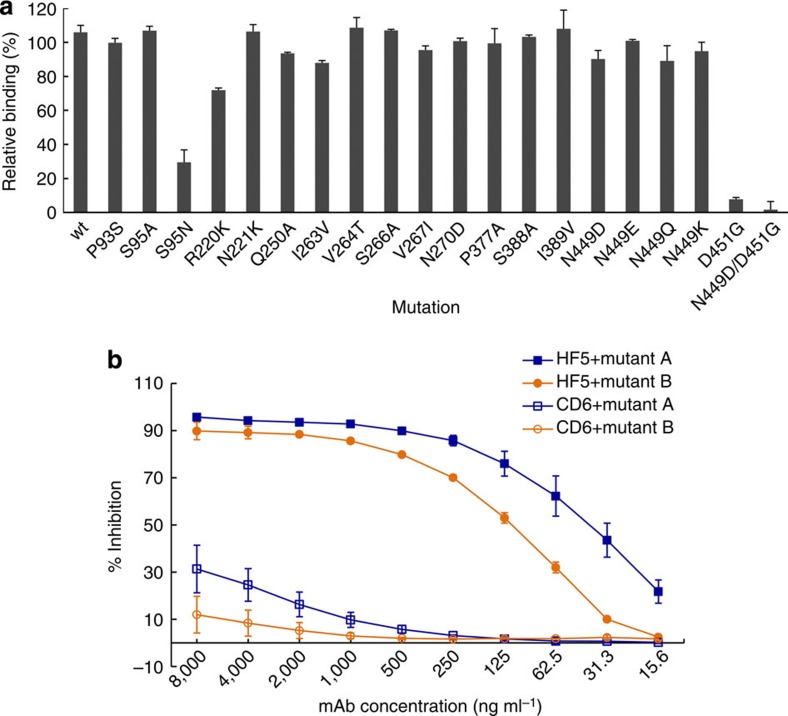
Amino acids in CA/09 NA critical for CD6 binding. (**a**) Binding of antibody CD6 to CA/09 NA mutants transiently expressed on 293T cells. ELISA signals generated with CD6 (1 μg ml^−1^) were normalized to those of mouse serum against CA/09 and were expressed as relative binding. Binding to wt CA/09 NA is shown in the first bar labelled wt. Each bar represents mean+s.d. of two independent assays performed in duplicate. (**b**) Inhibition of NA activity of two representative escape variants (mutant A and mutant B) selected in the presence of mAb CD6. The inhibition was measured with ELLA. Data are shown as mean±s.d. of three independent experiments.

Based on our structural analyses, site-directed mutagenesis was performed to construct a panel of CA/09 NA-expressing plasmids, with mutations at positions identified as potentially important for CD6 binding based on their surface exposure, charge and the comparison of the NA sequences of BR/07, CA/09 and VN/04. The impact of each NA mutation on CD6 binding was determined by ELISA, in which 293T cells transfected with mutant expression plasmids were used as antigen. Of the single-point mutations tested, those at positions 375 and 378 (W375A, W375G and N378A) did not support expression of NA, as examined with mouse serum against CA/09 (data not shown). The impact of substitutions at another 15 NA residues was determined by comparing the relative binding of CD6 to each mutant NA and the wild-type (wt) CA/09 NA. The S95N substitution decreased the binding of CD6 (ELISA signal (optical density (OD_490_)) with CD6<0.4) but not CA/09-specific mouse serum (OD_490_~1.2), whereas a conservative substitution (to Ala) at this position did not affect the binding (Fig. 5a; OD values with both CD6 and mouse serum were ~1.1, resulting in relative binding close to that of wt CA/09 NA). Although the hydroxyl group of Ser95 in NA does not interact directly with CD6, Ser95 contributes to the interaction with antibody through hydrophobic contacts with multiple residues (Thr28, Thr31 and Tyr32) of the HCDR1 loop (Supplementary Fig. 2a). Structural modelling suggests that a substitution with a bulky hydrophilic side chain such as Asn could potentially disrupt binding in this region, in particular the hydrogen bonds between Asn449 of the NA with Thr31 of the HCDR1 loop. Asn is present at position 95 in the NA of VN/04, partially explaining why CD6 cannot bind to this virus. Residues at positions 220, 221, 250, 263, 264, 267, 270 and 389 of the CA/09 NA were mutated to those present in BR/07 (Supplementary Table 2). Although R220K and I263V (Val263 is also present in VN/04 NA) mutations resulted in 34% and ~20% reduction in CD6 binding (Fig. 5a), respectively, no single mutation completely abolished binding. These data suggest that the inability of CD6 to bind BR/07 and VN/04 NA is determined by multiple interactions. In addition, we cannot exclude that differences in the quaternary structure of the NA tetramers of BR/07, CA/09 and VN/04 also play a role in determining the reactivity of CD6.

To identify mutations in NA that would allow replication of virus in the presence of CD6, escape variants were selected in eggs inoculated with a mixture of CD6 and H6N1_CA/09_ virus. Several rounds of selection were required before escape variants that resisted inhibition by CD6 in plaque assays were identified. All of the 12 variants selected had a double mutation, N449D and D451G, in the NA. As indicated by two representative variants (labelled as mutants A and B in Fig. 5b), the NA enzyme activity of these variants was still effectively inhibited by mAb HF5, but was resistant to inhibition by CD6 (Fig. 5b), suggesting that these two residues are critical for the interaction between CD6 and CA/09 NA. Indeed, in ELISA with NA expressed on 293T cells, D451G mutation reduced the relative binding of CD6 to below 10%, whereas the N449D/D451G double mutation abolished CD6 binding (Fig. 5a). In both the single and double mutants, the expression of NA was decreased but detectable with mouse serum (OD_490_~0.65), suggesting that these changes destabilize the NA protein. The NA with N449D and D451G double mutation was less efficient than the wt NA in cleaving the substrate (Supplementary Fig. 3), suggesting that its stability/activity was impaired. Interestingly, the N449D mutation alone did not have a big impact on CD6 binding. An N449K mutation, present in some recent pH1N1 isolates, did not significantly reduce CD6 binding either (Fig. 5a). These data suggest that residue 451 is a pivotal contact for CD6. Asp451 interacts directly with Tyr104 and indirectly, via a water molecule, with Arg100 on HCDR3 of CD6 (Supplementary Fig. 2a). A stereo view of the CD6 Fab/NA complex highlighting Asp451 is provided in Supplementary Fig.4. In addition, its main chain oxygen interacts with the hydroxyl of Thr215 in the neighbouring NA monomer. Thus, a mutation at this residue could affect mAb binding either directly or indirectly by destabilizing the NA dimer. Selection of the double N449D and D451G mutation in escape variants suggests a potential compensatory effect of N449D to stabilize the dimer since this latter mutation does not diminish CD6 binding on its own.

### CD6 inhibits NA activity by steric hindrance

**Figure 6:**
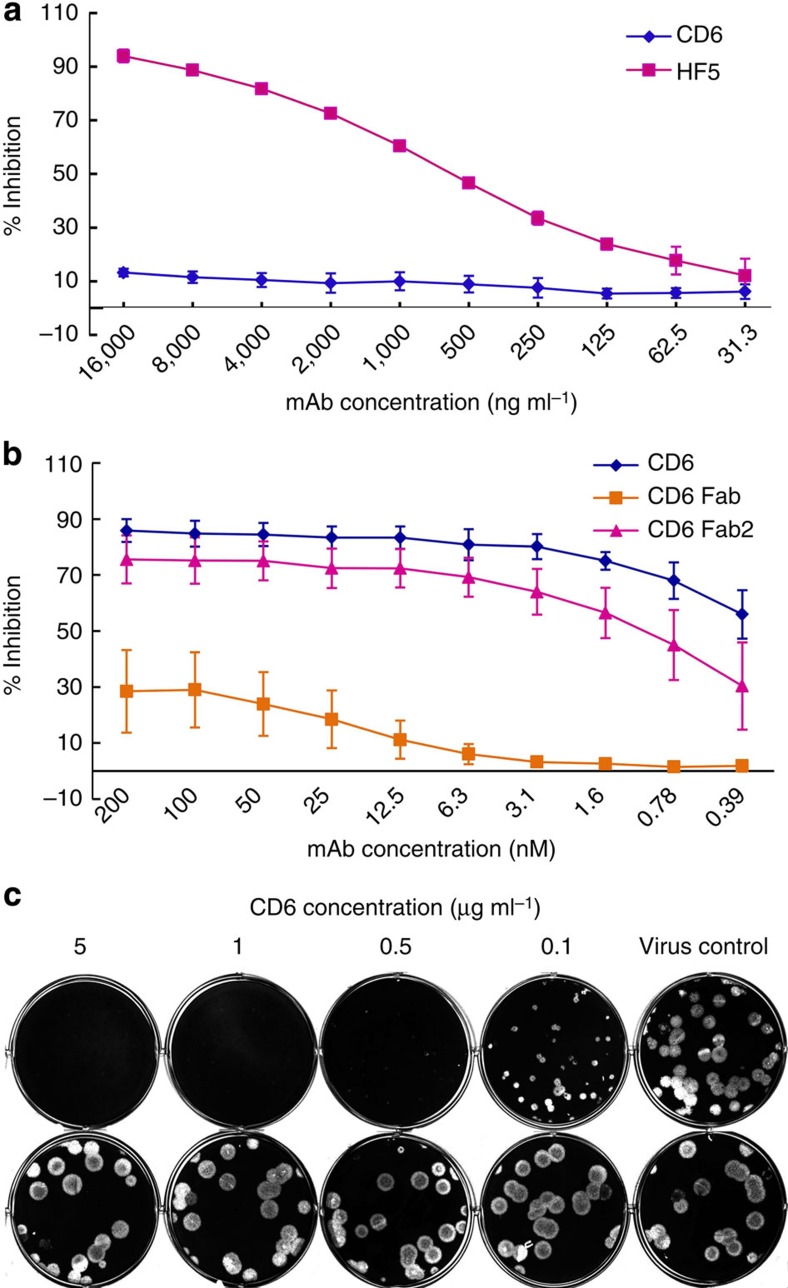
CD6 inhibits NA activity by blocking the access of substrate to the enzyme-active site. (**a**) Inhibition of CA/09 NA cleavage of a small substrate MU-NANA (MW: 489 Da) by mAbs CD6 and HF5. (**b**) Inhibition of CA/09 NA cleavage of a large substrate fetuin (MW: 49 kDa) by CD6 whole IgG, Fab2 and Fab. Data are shown as mean±s.d. of three independent experiments. The ability of the CD6 Fab, Fab2 and whole IgG to bind NA is shown in Supplementary Fig. 5. (**c**) CD6 inhibits CA/09 plaque formation when supplemented in the agar overlay (after virus infection of cells; upper panel), but does not inhibit plaque formation when supplemented in the virus inoculum (cells were infected with CA/09 virus pre-mixed with CD6 and after thorough washing, the infected cells were covered with agar without antibody; bottom panel).

As the X-ray crystallography showed that the binding by CD6 Fab did not change the conformation of NA, it is likely that CD6-mediated inhibition of NA is through steric hindrance, that is, CD6 blocks the access of substrate to the NA-active site. This hypothesis is supported by different experimental approaches. First, the inhibition of NA activity by CD6 was affected by the size of the substrate. Although CD6 inhibited the cleavage of fetuin (Fig. 1a), a large molecule with a molecular weight (MW) of 49 kDa, it did not inhibit NA from cleaving a small substrate 2′-(4-methylumbelliferyl)-α-D-*N*-acetylneuraminic acid (MU-NANA; MW: 489 D; Fig. 6a). This also confirms that CD6 binding does not have an allosteric effect on the enzyme-active site. Unlike CD6, antibody HF5 inhibited the cleavage of both fetuin and MU-NANA by CA/09 NA (Figs 1b and 6a). Second, the inhibition of NA activity by CD6 was impacted by the size of antibody. In assays using fetuin as substrate, the whole CD6 molecule (MW: 150 kDa) inhibited NA activity most efficiently, with slightly less inhibition by the CD6 Fab2 (MW: 110 kDa). However, there was little inhibition by the CD6 Fab (MW: 50 kDa) even at the highest tested concentration of 200 nM (10 μg ml^−1^; Fig. 6b). This low level of inhibition was not due to the lack of binding of NA by the CD6 Fab, as ELISA results showed similar binding of NA by Fab, Fab2 and the whole IgG, especially at higher concentrations (Supplementary Fig. 5). These data demonstrate that CD6 inhibits NA activity through steric hindrance rather than through structural distortion to inactivate the enzyme.

Because binding of NA antibody may also interfere with neighbouring HA molecules, we tested whether the bound CD6 molecule also hinders HA function. Pre-mixing CA/09 virus with CD6 before infecting MDCK cells did not affect the number and size of CA/09 plaques (Fig. 6c); however, when CD6 was supplemented in the agar overlay instead of being added to the virus inoculum, it inhibited the formation of CA/09 plaques (Figs 1c and 6c). This finding shows that CD6 inhibited virus release from infected cells, rather than blocking virus attachment and entry into cells.

### Amino-acid changes within the CD6 epitope are infrequent

To determine whether circulating pH1N1 viruses have emerged with changes in the CD6 epitope, we analysed all available pH1N1 NA gene sequences from GISAID (http://www.gisaid.org), excluding duplicates, those without full dates of collection or propagated in eggs. We found that since the emergence of pH1N1 virus in 2009, very few viruses with NA mutations at amino acids 95 (*n*=109; 1.5%) or 449 (*n*=38; 0.5%) have emerged (out of 7,958 available pH1N1 NA sequences). However, more viruses have been isolated with a substitution at Asp451 (*n*=196; 2.5%). The D451G mutation that reduces CD6 binding arose sporadically among several different NA lineages since 2009 (Supplementary Fig. 6), and was for a time fixed in the clade 6A subset of pH1N1 viruses. We performed a Bayesian molecular clock analysis to estimate the date of origin for the D451G mutation in the NA gene from clade 6 and showed that it arose in early 2010 (2010.026, Bayesian confidence interval, 2008.824–2010.929) along with an additional mutation N386S, within NA genetic clade 6A. Although Asn386 is a predicted glycosylation site, occupancy of this site was not apparent in the final structure. However, the B-factors for this residue are higher than its neighbouring residues, suggesting that this site may indeed be glycosylated. The D451G mutation was noticeable in the NA of viruses sequenced in 2012 (Supplementary Table 3) but was present in a much smaller proportion of sequences in 2013 (~3% of available pH1N1 NA sequences). The change in proportion of pH1N1 viruses with the D451G mutation corresponds with the decreased circulation of pH1N1 viruses from genetic group 6A since 2012. Approximately 95% of the viruses that circulated in 2013 contained NA of genetic clade 6B, which retains the CD6 epitope. To date, none of the NA sequences available in GISAID contain the N449D/D451G double mutation.

**Figure 7:**
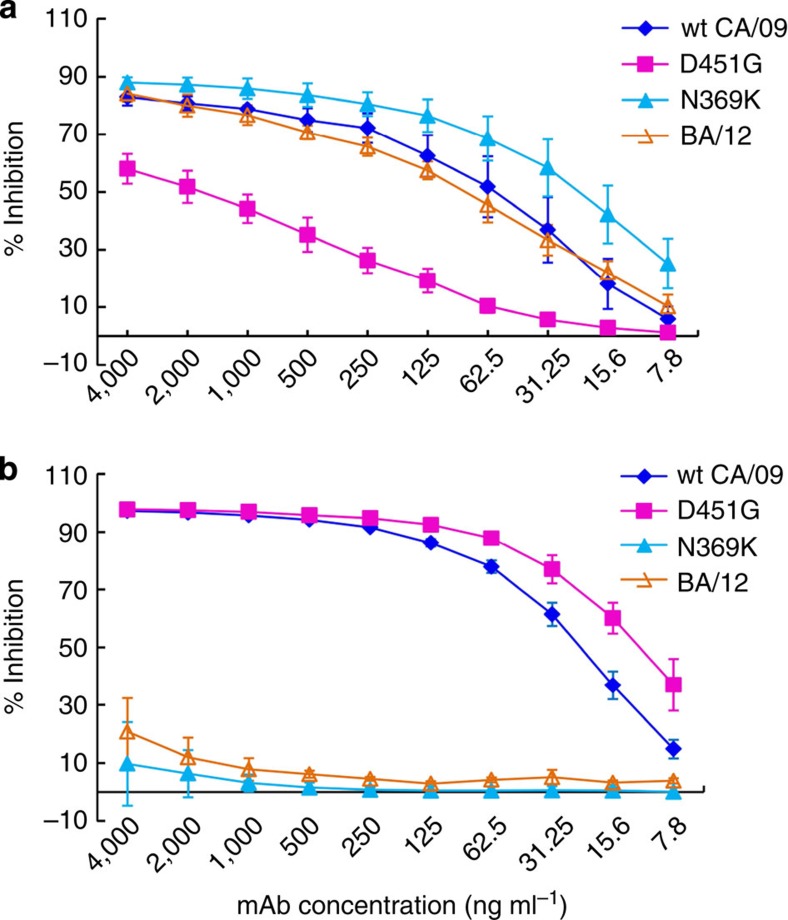
NA of genetic group 6A is sensitive to inhibition by CD6. (**a**) Inhibition of the NA activity of variant and circulating viruses by mAb CD6. The sensitivity of the NA of the following viruses was tested in ELLA: wt CA/09, CA/09 with single amino-acid mutation D451G or N369K in NA; BA/12 (genetic group 6A). BA/12 contains D451G mutation as well as additional mutations at residues 106, 241, 248, 369 and 386 in NA. (**b**) Inhibition of the enzyme activity of the NAs described in **a** by mAb HF5. Data are shown as mean±s.d. of three independent experiments.

Consistent with the greatly reduced CD6 binding to the D451G mutant NA of CA/09 observed in cell-based ELISA (Fig. 5a), this mutation alone resulted in significant loss of CD6’s ability to inhibit enzyme activity (Fig. 7a). Since this mutation is present in clade 6A NA of some viruses isolated in 2012 (Supplementary Fig. 6), we tested whether this clade of NA had become resistant to inhibition by CD6. We were surprised to observe that the NA of A/Bangladesh/2021/2012 (BA/12), a reference pH1N1 virus that has a clade 6A NA, retained sensitivity to CD6 inhibition (Fig. 7a). Besides the D451G mutation, BA/12 NA has additional changes, including V106I, V241I, N248D, N369K and N386S. It is not known how these additional changes help to retain the sensitivity of BA/12 NA to CD6 inhibition. Unlike CD6, the control mAb HF5 did not inhibit the NA of CA/09 when it was engineered to contain an N369K mutation, suggesting that HF5 recognizes an epitope with amino-acid 369 as one of the key contacts. This mutation is contained in the NA of BA/12 and therefore, as expected, BA/12 NA was not susceptible to HF5 inhibition (Fig. 7b). It is encouraging that CD6 remains effective against H1N1 viruses with variations in the CD6 epitope and the epitopes recognized by other antibodies (for example, the HF5 epitope); continued monitoring on the sensitivity of pH1N1 viruses to CD6 will help to determine whether CD6-resistant variants can occur in the future.

Although variants with amino acid changes within the CD6 epitope are infrequent, some pH1N1 strains have developed resistance to current antiviral drugs, including NA inhibitors^26^,^27^. We tested the ability of antibody CD6 to inhibit the NA activity of A/Bethesda/NIH107-D31/2009, a pH1N1 virus that is resistant to both oseltamivir and peramivir because of a H275Y mutation in the NA^4^,^27^. CD6 inhibited the drug-resistant virus at very low concentrations (IC_50_: 28±10 ng ml^−1^; Supplementary Fig. 7), highlighting the potential of the CD6 epitope to serve as a target for alternative antibody therapeutics against pH1N1 virus infection.

## Discussion

NA is the second most abundant surface glycoprotein on the influenza virus surface, and although its enzyme-active site has long been the focus for the design of antivirals, it can also be the target of therapeutic antibodies and novel vaccines. CD6, a NA-specific mAb, protects mice against lethal challenge with the reassortant pH1N1 virus CA/09-X179A, making it an attractive antibody candidate for treating infection with pH1N1 viruses, especially those resistant to licensed NA inhibitors. To fully understand its potential as a therapeutic agent, we elucidated the CD6 epitope by X-ray crystallography, and investigated the interactions that are most important for CD6 activity. Our data show that the CD6 epitope is unique, spanning neighbouring NA monomers, with 30 amino acids serving as antibody contacts.

Our results are interesting in that although antibody epitopes have previously been reported for N2 (ref. 28) and N9 (refs 22, 23, 29, 30) subtypes, this is the first structural report for a N1 NA/mAb complex. In addition, unlike the N2 and N9/Fab crystal structures that define epitopes on the upper face of individual NA monomers, the CD6 epitope differs by spanning the lateral face of neighbouring N1 monomers. Although an epitope present in intact dimers was also proposed for the N8 subtype-specific mAb (N8-4) based on analysis of mAb escape mutants^31^, the epitope was never structurally defined. Epitopes that span the monomers of NA and HA may be overlooked because of the use of screening assays that do not retain the quaternary protein structure. For HA, most of the mAbs reported, including those targeting conserved antigenic sites, bind to single HA1/HA2 monomers. Nevertheless, mAbs that bind simultaneously to two HA monomers have occasionally been described^32^,^33^, suggesting that the molecular structure of HA allows antibodies to be generated that bind across the monomer–monomer interface of the trimer.

The mAbs that span HA monomers are efficient in neutralizing virus *in vitro*^32^,^33^ and thus are likely to be protective *in vivo*. Our *in vivo* studies showed that NA-specific mAb CD6 protected mice against severe disease. When given prophylactically, a low dose of 0.5 mg kg^−1^ protected all mice against lethal CA/09-X179A virus challenge. In the therapeutic study, a single dose of 5 mg kg^−1^ or multiple doses of a lower dose (2.5 or 1 mg kg^−1^) protected mice from lethal virus challenge. These findings suggest that the interface of either HA or NA monomers can serve as an attractive target for the development of therapeutic antibodies against influenza.

The atomic structure of CA/09 NA in complex with CD6 Fab identified 30 amino acids that have contact with antibody. This number of contacts is far greater than those observed for other NA/Fab complexes—mAbs NC10 and NC41 have 14 and 17 amino-acid contacts in N9 (ref. 23), respectively—whereas mAb Mem5 contacts 17 amino acids in N2 (ref. 24). With a larger number of contacts between NA and mAb, it might be less likely for single mutations in NA to eliminate mAb binding, even though the antibody avidity may change to some degree. In the case of CD6, it required multiple rounds to select escape mutant viruses. These escape variants bear double mutations N449D/D451G in NA, rather than single amino-acid mutation as often observed in mutant selection with other NA mAbs^15^,^34^,^35^. Interestingly, residues 449 and 451, together with residue 95, are present as a cluster and therefore possibly form a pivotal anchor for CD6 binding.

After several years of circulation in the human population, the epitope targeted by CD6 remains largely conserved, although mutations at some of the residues in CD6 epitope have occurred at a low rate. None of the pH1N1 NA sequences available in GISAID contain both N449D and D451G, the mutations identified in CD6 escape variants. A small proportion of pH1N1 viruses contain the D451G single substitution in NA. This single change in the NA of CA/09 reduced CD6 binding; however, additional mutations in the NA might have helped some of these variant viruses to retain sensitivity to CD6, as demonstrated by our analysis with a representative virus BA/12.

The findings that CD6 inhibited NA cleavage of large molecule fetuin but not the small substrate MU-NANA suggested that this antibody inhibited NA activity by blocking access of substrate to the enzyme-active site. This concept is in agreement with the structural observations; there was no distortion of the NA structure in complex with CD6, and although the boundary of the CD6 epitope, formed by the HCDR2 loop, is distant from the NA-active site, amino-acid contacts for the L chain on the neighbouring monomer are in close proximity to the active site. Thus, CD6 is likely to block the active site of the monomer to which the L chain binds, with four CD6 molecules potentially needed to inhibit all NA-active sites of a tetramer. Another observation that supports a mechanism of steric hindrance is that the size of the CD6 molecule impacts inhibition. In our *in vitro* assays, CD6 Fab2 inhibited CA/09 NA to a greater degree than CD6 Fab, and the best inhibition was observed with whole CD6 IgG. In the case of the interaction between NA and the whole CD6 molecule, the other Fab arm as well as the Fc region of the antibody may contribute to blocking access of substrate to NA, either by obstructing the active site by its bulk, or by cross-linking adjacent NA tetramers.

In conclusion, this is the first report of the structure of NA subtype N1 in complex with a highly protective mAb, and it is the first structural analysis of an epitope that spans adjacent NA monomers. The large number of interactions between NA and CD6 are likely to stabilize the complex. The apparent need for two amino-acid changes in CD6 epitope to enable antibody escape consequently reduces the likelihood of generating CD6-resistant variants. The absence of NA sequences with changes at both 449 and 451 residues gives confidence that CD6-resistant viruses are not currently in circulation. These observations show that mAb CD6 is suitable as a prophylactic or therapeutic treatment against H1N1 influenza, and is of particular value in case of emerging NA inhibitor-resistant variants. Our results provide a rationale for the development of novel vaccines and therapeutic antibodies that target similar epitopes in NAs of other influenza types and subtypes, including those with pandemic potential.

## Methods

### Viruses

Viruses used in this study include: pH1N1 viruses wt CA/09, CA/09-X179A, a high growth reassortant of CA/09 that has NA sequence identical to that of the wt parent virus, and a drug-resistant H1N1 virus A/Bethesda/NIH107-D31/2009; seasonal H1N1 virus BR/07; an attenuated H5N1 VN/04 virus (ΔVN/04) that contains the HA and NA genes of VN/04 (polybasic residues in the cleavage motif of HA deleted) and the internal genes of A/Puerto Rico/8/1934; reassortant H6N1 viruses H6N1_CA/09_, H6N1_BR/07_, H6N1_VN/04_ and H6N1_BA/12_, viruses that contain the HA gene of H6N2 virus A/Turkey/Massachusetts/3740/1965 and the NA gene of CA/09, BR/07, VN/04 or BA/12; H6N1_CA/09_ viruses, which contain N369K or D451G single mutation in the NA. Primers used to clone and sequence NA genes are listed in Supplementary Table 4. Viruses were grown in 10-day-old embryonated chicken eggs and, if necessary, were inactivated with β-propiolactone (Sigma-Aldrich) and purified by sucrose gradient centrifugation.

### Generation of mouse hybridomas secreting NA-specific mAbs

Hybridomas were generated following a published procedure^15^. Briefly, female BALB/c mice (6-week-old, Jackson Laboratory) were immunized and boosted with CA/09. Splenocytes from an immunized mouse were fused with Sp2/0 cells (obtained from the Centers for Disease Control and Prevention, Atlanta, GA, USA). Hybridomas were screened in cell-based ELISA with NA expressed on 293T cells transfected with pCAGGS-CA/09NA, a plasmid that contains the NA gene of CA/09 (ref. 36). Positive hybridomas were subcloned twice by limiting dilution. Antibodies were purified from serum-free tissue culture supernatants using protein G columns (GE Healthcare) and isotyped with the Pierce rapid isotyping kit (Pierce Biotechnology). The H and L chain genes of mAb CD6 were sequenced by ProMab Biotechnologies, Inc. and Syd Labs, Inc.

### Preparation of Fab and Fab2

Purified IgG (1 mg) was digested to Fab and Fc fragments by exposure to 40 μg activated papain (Sigma-Aldrich) in a solution containing 10 mM cysteine, 100 mM sodium acetate pH 5.5, 125 μM EDTA for 2–4 h at 37 °C. Fab2 was produced by digestion with pepsin (Sigma-Aldrich) for 2–4 h at 37 °C. Fab and Fab2 fragments were subsequently separated from Fc by affinity chromatography using a protein A Hi-trap column (GE Healthcare) and purified to homogeneity by size exclusion chromatography (SEC)^37^. SDS–polyacrylamide gel electrophoresis was performed to confirm the sizes of Fab and Fab2. The NativePAGE Novex Bis-Tris gel, SimplyBlue SafeStain staining solution and protein standard were purchased from Invitrogen, Inc.

### CA/09 NA expression and purification

Recombinant CA/09 NA protein was expressed in a baculovirus expression system with an additional amino-terminal cassette containing a His-tag, a tetramerization domain from the human vasodilator-stimulated phosphoprotein and a thrombin cleavage site^38^. Secreted protein was recovered from the culture supernatant and purified by metal affinity chromatography and SEC. For structural analyses, the protein was further subjected to thrombin cleavage and SEC.

### Formation and purification of the CD6 Fab/NA complex

CD6 Fab was mixed with purified, His-tag depleted, recombinant CA/09 NA monomer at a 1:1 molar ratio of Fab to NA. The resulting CD6 Fab/NA complex was purified away from unbound substrates by SEC in a buffer comprising 50 mM Tris-HCl (pH 8.0) and 150 mM NaCl. The CD6 Fab/NA complex eluted as a single peak between the 158 and 670 kDa molecular weight markers (data not shown) and was concentrated to 15 mg ml^−1^ for subsequent crystallization trials.

### Crystallization and data collection of CD6 Fab/NA complexes

Initial sparse-matrix crystallization screening was carried out using a Topaz Free Interface Diffusion Crystallizer system (Fluidigm Corporation). Hits were obtained after 24 h in several conditions containing the precipitant polyethylene glycol variables. Following optimization and translation to sitting drop vapour diffusion, diffraction quality crystals were obtained at 23 °C against a reservoir containing 0.09 M malonic acid, 0.013 M ammonium citrate tribasic, 0.006 M succinic acid, 0.015 M DL-malic acid, 0.02 M sodium acetate, 0.025 M sodium formate, 0.008 M ammonium tartrate dibasic, 0.1 M HEPES/NaOH, and 10% (w/v) PEG 8000, final pH 7.0 (condition 68 from the MCSG-3 screen, Microlytic North America). The CD6 Fab/NA complex data set was collected from a single crystal to 2.8 Å resolution at the Advanced Photon Source SER CAT 22-ID beamline (wavelength 1.0 Å). Data were processed with DENZO-SCALEPACK suite^39^.

### Structure determination and refinement

The CD6 Fab/NA complex structure was determined at 2.8 Å resolution by the molecular replacement method using Phaser^40^. A strong solution was obtained for the NA component of the complex using a single monomer of NA (PDB: 4B7M^21^). An initial search for CD6 Fab determined the position of the variable (Fv) domains using the Fv component of a mouse IgG_1_ Fab (PDB: 1WEJ^41^) as a search model. A subsequent search using the constant (Fc) domains of a mouse IgG_2a_ Fab (PDB: 3FO9 (ref. 42)) located the Fc region. Two NA monomers and two Fabs occupy the asymmetric unit.

Rigid-body and restrained refinement of the molecular replacement solution was carried out using REFMAC5 (ref. 43) and model building was performed in Coot^44^. 2F_o_−F_c_ electron density was well-defined throughout the model. Restrained refinement of the structure was completed in REFMAC5 using TLS refinement^45^. The final model was assessed using MolProbity^46^. In the final model, 95% of the residues were in favoured regions of Ramachandran plot, with 5% in additional allowed regions.

### Plaque assay

Confluent MDCK cells (originally obtained from ECACC) in six-well plates were inoculated with diluted virus. After 1 h incubation at 37 °C, cells were overlaid with agar supplemented with mAb at indicated concentrations. Cells inoculated with virus and overlaid with agar without mAb were set up as controls. When examining the effect of antibody CD6 on virus entry, CA/09 virus was pre-mixed with CD6 and incubated for 1 h at 37 °C before inoculation of cells. After 1 h incubation of cells with the inoculum, cells were washed with PBS to remove CD6, and then covered with agar without antibody. On day 3 post virus inoculation, cells were stained with crystal violet solution to visualize plaques.

### Site-directed mutagenesis

Nucleotide changes corresponding to a panel of single amino mutations and a double mutation were introduced into the CA/09 NA gene in pCAGGS-CA/09NA plasmid with QuickChange multisite-directed mutagenesis kit (Stratagene). Mutant plasmids were sequenced to verify the presence of introduced mutations and the absence of additional, unwanted mutations. Primers used in site-directed mutagenesis are listed in Supplementary Table 4.

### Cell-based ELISA

CA/09 NA and its mutants were expressed on 293T cells (kindly provided by Hang Xie, Center for Biologics Evaluation and Research, Food and Drug Administration, Silver Spring, MD, USA) by transfecting with wt or mutant pCAGGS-CA/09NA plasmids using Lipofectamine 2000 reagent (Invitrogen). ELISA was performed with the transfected cells as described previously^15^. Briefly, transfected cells were fixed with glutaraldehyde and blocked with 3% BSA in PBS. Cells were then incubated with purified mAbs or antiserum, followed by incubation with horse radish peroxidase-conjugated goat-anti-mouse IgG (Sigma-Aldrich). The signal was developed using *O*-phenylenediamine dihydrochloride as substrate. The reaction was stopped with sulfuric acid and OD_490_ values were read. Hyperimmune mouse serum against CA/09-X179A virus (with hemagglutination inhibition titer ≥320) was used as a positive control and for examining the expression of NA. In assays to identify critical amino acids for mAb binding, mutations (W375A, W375G and N378A) that resulted in the absence of binding by mouse serum (OD_490_<0.3) were not included in further analysis. For all other NAs (mutant and wt), the signals generated by mAb binding to each NA were normalized to those generated by mouse serum (the background signals generated with mock-transfected cells were subtracted from both the mAb and mouse serum signals) and therefore expressed as relative binding.

### NA inhibition assay

The inhibition of NA enzyme activity by mAbs was measured by ELLA in a 96-well plate format^47^. Briefly, mixtures of virus and serial dilutions of antibody were incubated in duplicate wells of a fetuin-coated plate. After overnight incubation at 37 °C, the plates were washed and then incubated with peanut agglutinin conjugated to horse radish peroxidase. After 2 h incubation at room temperature, the plates were washed and substrate added. The reaction was stopped 10 min later and the OD read at 490 nM. The IC_50_ was determined by quadratic curve fitting (GraphPad Prism).

### Selection of mAb escape variants

Escape variants were selected as previously reported^15^,^48^. mAb CD6 was mixed with 10^6^ plaque forming units of H6N1_CA/09_ virus and inoculated into 10-day-old embryonated chicken eggs. This reassortant virus was elected for generation of escape variants in order to keep the use of H6 reassortant viruses consistent with that used in subsequent ELLA. As the initial selection did not result in escape variants that resisted inhibition by CD6 in plaque assay, further selections were performed. Briefly, allantoic fluid (P1) from the initial selection was pooled and inoculated into eggs to allow the expansion of residual parent virus (if any) and potential variants. Allantoic fluid collected from these eggs (P2) was pooled, mixed with mAb CD6 and inoculated into eggs again. This process was repeated two more times, and the resultant P5 allantoic fluid was collected from individual eggs and tested in plaque assay. Three large plaques that formed in the presence of CD6 were picked and expanded in eggs. P6 viruses (expanded from each single plaque) were characterized and sequenced. The NA gene was amplified by reverse transcription–PCR^49^ and PCR products were sequenced at the core facility of Center for Biologics Evaluation and Research, Food and Drug Administration.

### Prophylactic and therapeutic studies

All mouse studies followed protocols approved by the Institutional (Center for Biologics Evaluation and Research, Food and Drug Administration) Animal Care and Use Committee and followed federal guidelines. Female DBA/2 mice (7-week-old, five mice per cage) were received from the Jackson Laboratory and housed with food and water supplied *ad libitum*. Cages were randomly assigned to experimental groups composed of 15 mice, a number adequate to demonstrate differences in survival (*n*=10) and lung virus titres (*n*=5). To examine the prophylactic efficacy of CD6, mAb was administered i.p. 12 h before intranasal challenge with 10 LD_50_ of CA/09-X179A. On day 3 p.c., five mice in each group were euthanized and the lungs were collected for virus titration in MDCK cells. To examine the therapeutic efficacy, groups of DBA/2 mice (*n*=10) were treated with CD6 after virus infection. In all experiments, body weight and mortality were monitored for up to 14 days p.c. Mice that were moribund or lost ≥25% weight were euthanized. The persons monitoring experimental animals but not the primary investigator were blinded to group allocation.

### Phylogenetic analyses

pH1N1 NA gene sequences available from the GISAID Epiflu database (http://www.gisaid.org) excluding duplicates, those without full dates of collection or propagated in eggs (*n*=7,958) were aligned using MAFFT^50^. Neighbour-joining phylogenetic tree of all pH1N1 NA genes was constructed in MEGA version 5 using Tamura-Nei nucleotide substitutions model^51^. For Bayesian molecular clock analysis, a random sampling of ten viruses per year since 2009 were added to a subset of viruses with mutations at NA amino-acid residues 95, 449 or 451 and the current vaccine strain, CA/09, for a final data set of 182 taxa. Temporal phylogenies were inferred using a log normal distribution-relaxed molecular clock with the SRD06 substitution model in BEAST v1.7.0 using Bayesian ancestral state reconstruction^52^. Chain lengths of 150 million steps were used with a 10% burn-in removed in two independent runs.

## Author contributions

H.W., J.S. and M.C.E. designed the experiments; H.W., H.Y., D.A.S., R.J.G., L.C., J.G., L.J. and P.J.C. performed the experiments; H.W., H.Y., D.A.S., R.J.G., J.V., J.S. and M.C.E. analysed the data; H.W., H.Y., R.J.G., J.S. and M.C.E. wrote the paper. All authors read and approved the manuscript.

## Additional information

**Accession codes:** Coordinates and structure factors for CD6 Fab/NA complex have been deposited in the Protein Data Bank under accession code 4QNP.

**How to cite this article**: Wan, H. *et al*. Structural characterization of a protective epitope spanning A(H1N1)pdm09 influenza virus neuraminidase monomers. *Nat. Commun.* 6:6114 doi: 10.1038/ncomms7114 (2015).

## Supplementary Material

Supplementary Information Supplementary Figures 1-7, Supplementary Tables 1-4 and Supplementary References.

## Figures and Tables

**Table 1:** Data collection and refinement statistics.

	**CD6-CA/09 NA**
*Data collection*	
Space group	C222
Cell dimensions	
*a*, *b*, *c* (Å)	141.74, 195.78, 141.92
*α*, *β*, *γ* (°)	90, 90, 90
Resolution (Å)	50–2.80 (2.87–2.80)*
*R*_sym_ or *R*_merge_	0.143(0.521)
*I*/σ*I*	18.1(4.4)
Completeness (%)	93.5(99.1)
Redundancy	4.7(5.0)
	
*Refinement*	
Resolution (Å)†	50–2.80 (2.87–2.80)
No. of reflections	46,132
*R*_work_/*R*_free_	0.181/0.224
No. of atoms	
Protein	12,635
Ligand/ion	116
Water	181
*B*-factors	
Protein	48.1
Ligand/ion	82.0
Water	40.1
r.m.s. deviations	
Bond lengths (Å)	0.013
Bond angles (°)	1.60

^*^Values in parentheses are for highest-resolution shell.

^†^A single crystal was used for both structure determination at 2.8 Å resolution and refinement.
