# Supplementary material for: Structural characterization of a protective epitope spanning A(H1N1)pdm09 influenza virus neuraminidase monomers
Source: Nat Commun. 2015 Feb 10;6:6114. doi: 10.1038/ncomms7114 (PMC4347215; doi:10.1038/ncomms7114)
Supplement: Supplementary Information — Supplementary Figures 1-7, Supplementary Tables 1-4 and Supplementary References. [file ncomms7114-s1.pdf]

SUPPLEMENTARY INFORMATION

Structural characterization of a protective epitope spanning A(H1N1)pdm09 influenza virus neuraminidase monomers

Hongquan Wan, Hua Yang, David A. Shore, Rebecca J. Garten, Laura Couzens, Jin Gao, Lianlian Jiang, Paul J. Carney, Julie Villanueva, James Stevens, Maryna C. Eichelberger

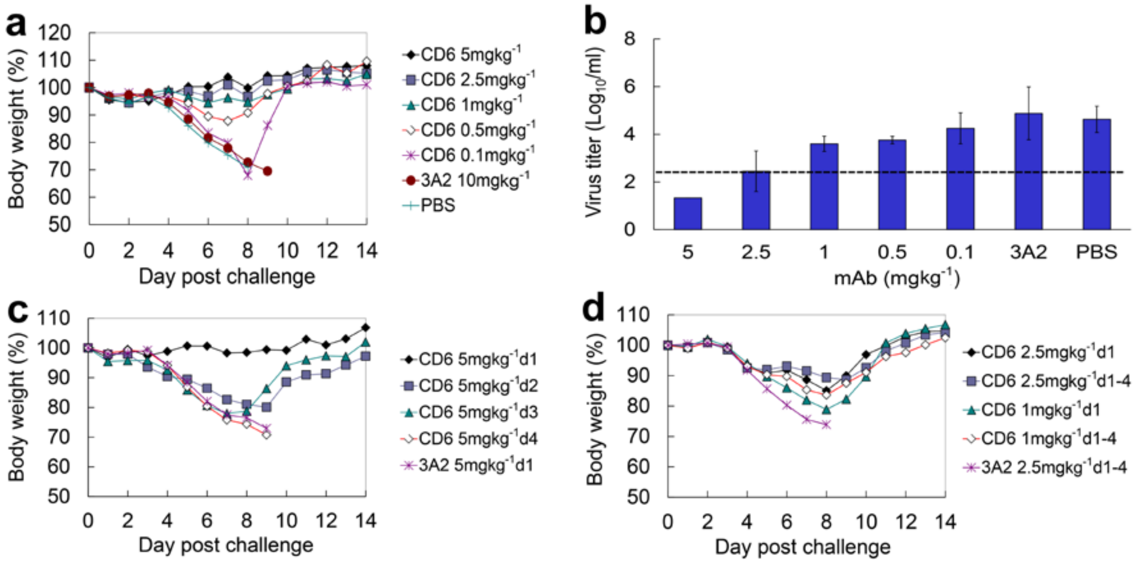

Supplementary Figure 1: Clinical and virologic evidence of CD6 efficacy in prophylactic and therapeutic studies. (**a**) Weight loss of mice (n=10) in prophylactic studies. Female DBA/2 mice were injected i.p. with mAb CD6 12 h before challenge with 10 LD_{50} of CA/09-X179A. The survival curves are shown in **Figure 2a** of the main text. (**b**) Lung virus titers of mice (n=5) in prophylactic studies. Mice were treated with CD6 and infected as described in (**a**) and then euthanized on day 3 p.c. The lungs were collected and homogenized for virus titration in MDCK cells. The dotted line denotes the detection limit (2.2 $Log_{10}TCID_{50}$ ml^{-1}). A titer of 1.7 $Log_{10}TCID_{50}$ ml^{-1} was arbitrarily set to represent titers below the detection limit. (**c**) Weight loss of mice (n=10) in single dose therapeutic studies. Female DBA/2 mice were infected intranasally with 10 LD_{50} of CA/09-X179A and 5 mg kg^{-1} of mAb CD6 was administered i.p. 1, 2, 3 or 4 days later. (**d**) Weight loss of mice (n=10) treated therapeutically with single or multiple doses of CD6. As described for (**c**), DBA/2 mice were infected with CA/09-X179A and treated with either 2.5 or 1 mg kg^{-1} CD6 24 h later (d1) only or once daily (24 h intervals) for 4 days (d1-4). The survival curves for these experiments are shown in **Figures 2b and 2c** of the main text.

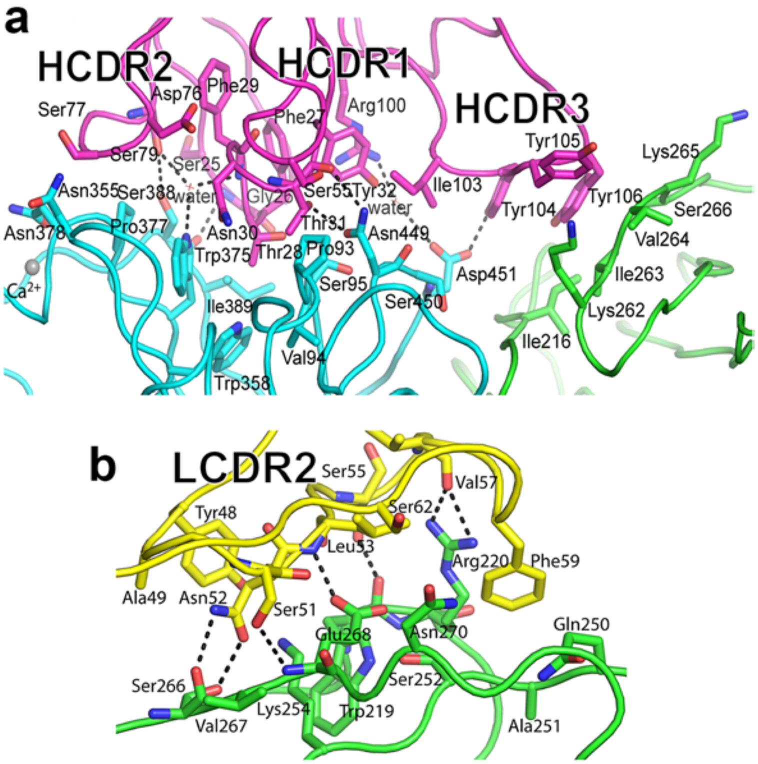

Supplementary Figure 2: Detailed analysis of the CD6 antibody interface with the NA. (a) The interface of H chain and CA/09 NA dimer. Waters that mediate interactions at the interface are highlighted as red crosses. (**b**) The interface of LCDR2 and the CA/09 NA monomer. Residues key to the antibody association are highlighted in sticks and hydrogen bonds are shown as dashed lines. Interactions were analyzed using MONSTER[1], PISA[2] and HBPLUS[3].

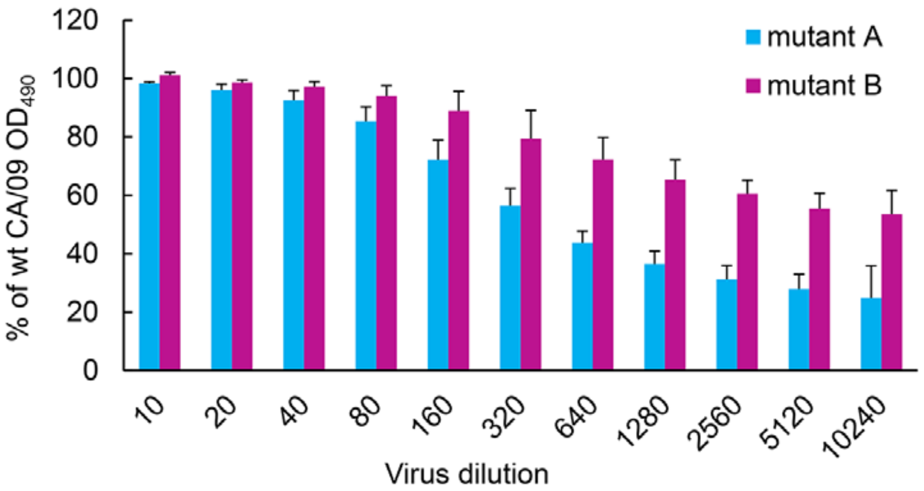

Supplementary Figure 3: The NAs of escape mutants selected by mAb CD6 are less efficient than wt CA/09 NA. Enzyme activity was measured with fetuin (MW: 49 kD) as substrate in ELLA, as described in Methods. Briefly, two representative escape mutants (mutants A and B, see the legend to **Figure 5**) and virus containing wt CA/09 NA, were normalized by hemagglutination assay to have the same titer (1024), then serially diluted and added to 96-well plates coated with fetuin, followed by incubation overnight at 37 °C; the plates were washed and then incubated with peanut agglutinin conjugated to horse radish peroxidase to detect the exposed galactose due to the removal of sialic acid from fetuin by NA; the signal was developed using o-phenylenediamine dihydrochloride (OPD) as substrate. The OD values generated with mutants were expressed relative to those obtained at the same dilution as wt CA/09 NA. Data are shown as mean ± s.d. of three independent experiments.

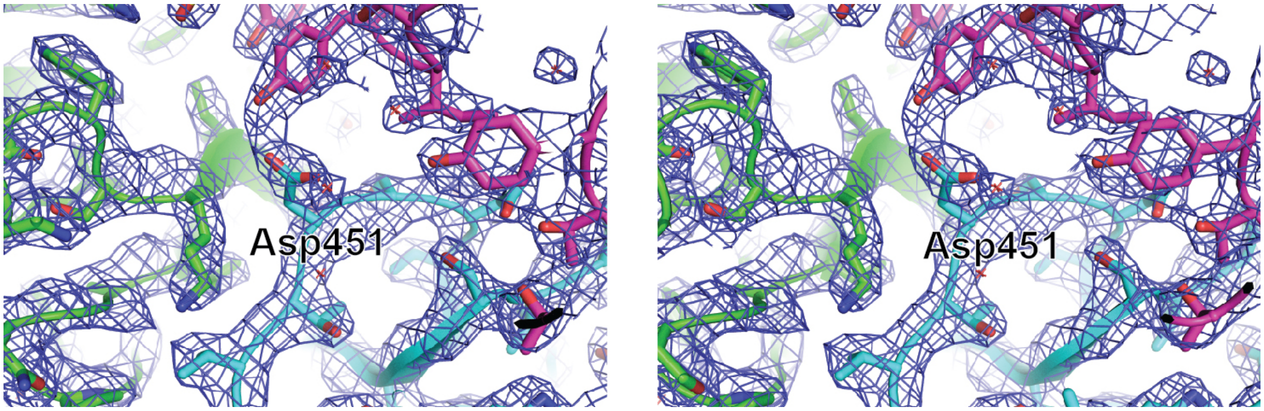

Supplementary Figure 4: Stereo view of the CD6 antibody interface (pink) with Asp451 of the CA/09 NA dimer (cyan and green). The 2Fo−Fc electron density map contoured at 1.0 σ is shown in blue.

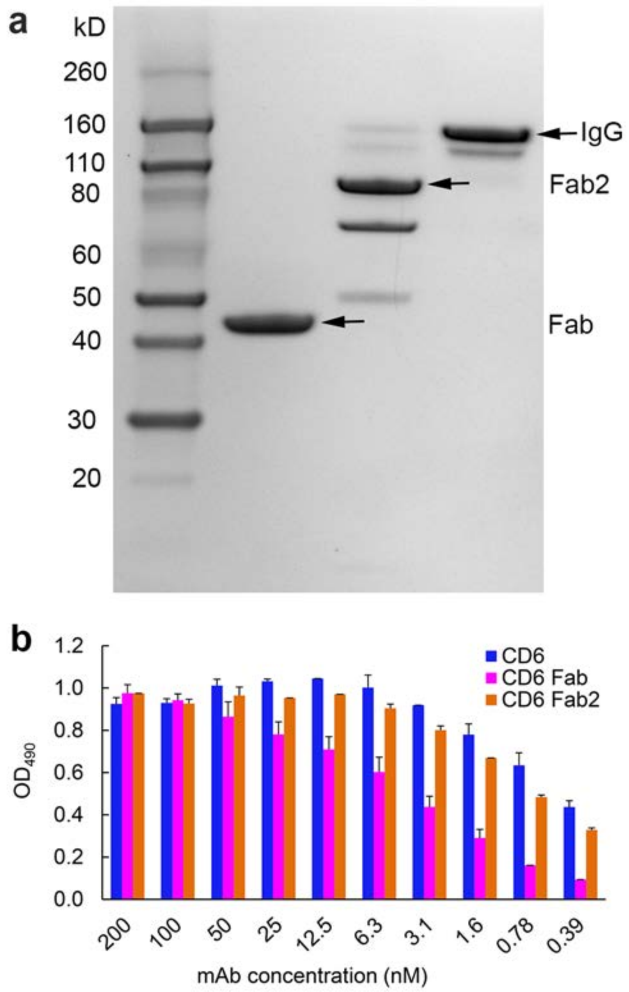

Supplementary Figure 5: CD6 Fab and Fab2 retain ability to bind NA. (**a**) SDS-PAGE of CD6 Fab (lane 2), Fab2 (lane 3) and whole IgG (lane 4). Lane 1: Novex sharp pre-stained protein standard (Invitrogen, Carlsbad, CA, USA; catalog number: LC5800). Lanes 2-4: CD6 Fab, CD6 Fab2 and whole IgG. (**b**) Binding of CD6 Fab, Fab2 and the whole IgG to CA/09 NA measured in ELISA. HRP conjugated goat-anti-mouse IgG (Fab specific) (Sigma-Aldrich, St. Louis, MO, USA; catalog number: A2304) was used to detect the bound antibody. Each bar represents mean + s.d. of two independent assays performed in duplicate.

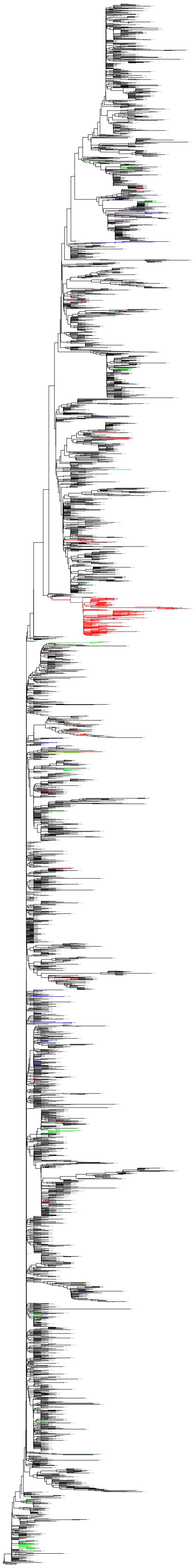

Supplementary Figure 6: Phylogenetic tree of pH1N1 NAs. The tree was constructed with n=7958 pH1N1 NA gene sequences available from the GISAID Epiflu database (www.gisaid.org). Since the phylogenetic tree includes analysis of a large number of NA sequences, color annotation was used to point out the relatively few viruses carrying amino acid changes in NA that may contribute to CD6 binding; a magnification 3200-fold allows the annotation and name of each strain to be read. Amino acid changes within the CD6 epitope are annotated on the phylogenetic tree as follows: NA sequences that have changes at residue 95 are shown as green diamonds; changes at residue 449 are shown as blue triangles; changes at residue 451 which do not have D451G in the sequence are shown as open red circles; changes of D451G alone are shown as closed red circles; and changes that include D451G and N386S are shown as closed red squares.

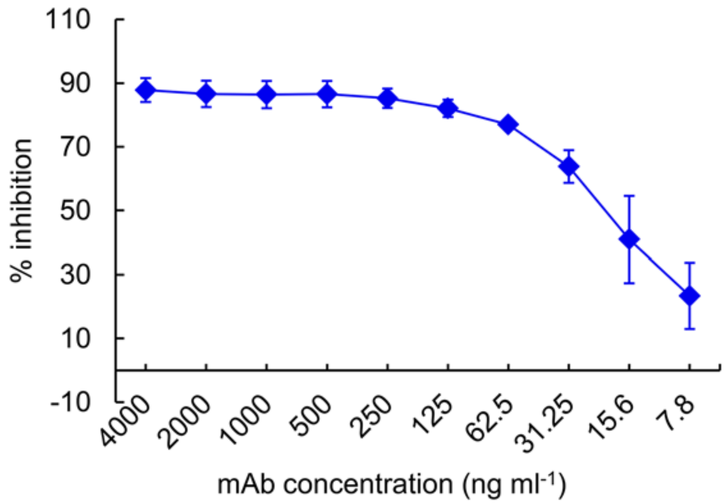

Supplementary Figure 7: Inhibition of the NA activity of A/Bethesda/NIH107-D31/2009 virus by antibody CD6. The pH1N1 virus has a H275Y mutation in the NA, and therefore is resistant to NA inhibitors oseltamivir and peramivir. The inhibition was measured with ELLA, in which fetuin (MW: 49 kDa) was used as substrate. Serial dilutions of antibody CD6 were added to wells prior to the addition of virus. Data are shown as mean ± s.d. of three independent experiments.

Supplementary Table 1 Contacts between buried surface residues of CD6 and CA/09 NA

NA monomer A	NA monomer B	Type of contact	Light chain	Heavy chain
Pro93		Hydrophobic<br>Hydrophobic		Thr31<br>Ser55
Val94		Hydrophobic<br>Water<br>Hydrophobic		Thr28<br>Asn30<br>Thr31
Ser95		Hydrophobic		Thr28,Thr31,Tyr32
	Ile216	Hydrophobic		Tyr106
	Lys217	Water		Tyr104
	Trp219	Hydrogen bond	Ser55	
	Arg220	Hydrogen bond<br>Hydrophobic	Val57<br>Phe59	
	Asn221	Hydrogen bond	Ser55	
	Gln250	Hydrophobic	Phe59	
	Ala251	Hydrophobic	Phe59	
	Ser252	Hydrophobic	Phe59	
	Lys254	Water<br>Hydrophobic	Tyr48<br>Asn52	Tyr104
	Lys262	Hydrophobic		Tyr105
	Ile263	Hydrophobic		Tyr105/Tyr106
	Val264	Hydrophobic		Tyr105/Tyr106
	Lys265	Hydrophobic		Tyr106
	Ser266	Hydrophobic<br>Water<br>Hydrogen	 Ala49<br>Asn52	Tyr106
	Val267	Hydrophobic	Ser51	
	Glu268	Hydrogen<br>Hydrophobic<br>Hydrogen	Ser51<br>Asn52<br>Leu53	
	Asn270	Hydrophobic	Ser62	
Asn355		Hydrophobic		Ser77
Trp358		Hydrophobic		Thr28
Trp375		Water<br>Water<br>Hydrophobic<br>Hydrophobic<br>Hydrophobic		Phe29<br>Ser79<br>Phe27<br>Thr28<br>Asn30
Pro377		Hydrophobic<br>Hydrophobic		Ser77<br>Asp76
Asn378		Hydrophobic		Ser77
Ser388		Hydrogen<br>Hydrogen<br>Hydrophobic<br>Hydrophobic<br>Hydrophobic		Gly26<br>Ser79<br>Ser25<br>Gly26<br>Phe27
Ile389		Hydrophobic<br>Hydrophobic<br>Hydrophobic		Gly26<br>Phe27<br>Thr28
Asn449		H bond<br>Hydrophobic<br>Hydrophobic		Thr31<br>Thr31<br>Ile103
Ser450		Hydrophobic		Ile103
Asp451[a]		H bond<br>Water<br>Hydrophobic		Tyr104<br>Arg100<br>Tyr104

[a]Asp451 forms an H-bond with Thr215 of the neighboring NA monomer.

Supplementary Table 2 Comparison of the 30 residues in the CD6 epitope to those in the NA of BR/07 and VN/04 viruses

Virus	Position/residue														
	93	94	95	216	217	219	220	221	250	251	252	254	262	263	264
CA/09	P	V	S	I	K	W	R	N	Q	A	S	K	K	I	V
BR/07	S	I	.[a]	.	.	.	K	K	A	.	.	.	.	V	T
VN/04	.	I	N	.	.	.	.	.	.	.	.	.	.	V	.

Supplementary Table 2-continued

Virus	Position/residue														
	265	266	267	268	270	355	358	375	377	378	388	389	449	450	451
CA/09	K	S	V	E	N	N	W	W	P	N	S	I	N	S	D
BR/07	.	.	I	.	.	.	.	.	.	.	.	V	.	.	.
VN/04	.	.	.	.	D	.	.	.	.	.	.	V	.	.	.

[a]Residues identical to those in CA/09 NA are shown as dots.

Supplementary Table 3 Variation of CD6 epitope residues in pH1N1 viruses

Residue	Mutation rate by year % (number of pH1N1 isolates with mutation)[a]					Total
	2009	2010	2011	2012	2013	
Pro93	0.05 (2)	0.5 (6)	0.84 (9)	0.16 (1)	1.22 (10)	0.35 (28)
Val94	0.02 (1)	0.08 (1)	0.09 (1)	0.33 (2)	0.24 (2)	0.09 (7)
**Ser95**[b]	2.13 (91)	0.67 (8)	0.28 (3)	0.66 (4)	0.37 (3)	1.37 (109)
Ile216	0.16 (7)			0.33 (2)	0.24 (2)	0.14 (11)
Lys217	0.07 (3)	0.17 (2)		0.82 (5)	0.12 (1)	0.14 (11)
Trp219			0.09 (1)			0.01 (1)
Arg220	0.23 (10)	0.67 (8)	1.49 (16)	1.48 (9)	8.19 (67)	1.39 (111)
Asn221	0.07 (3)	0.25 (3)		0.66 (4)	0.24 (2)	0.15 (12)
Gln250		0.08 (1)				0.01 (1)
Ala251	0.02 (1)					0.01 (1)
Ser252						
Lys254		0.08 (1)				0.01 (1)
Lys262	0.09 (4)	0.08 (1)	0.28 (3)		0.12 (1)	0.11 (9)
Ile263	0.28 (12)	0.76 (9)	0.19 (2)	0.33 (2)	0.12 (1)	0.33 (26)
Val264	0.23 (10)	2.27 (27)	2.52 (27)	0.99 (6)	1.34 (11)	1.03 (82)
Lys265	0.02 (1)	0.08 (1)	0.09 (1)		0.24 (2)	0.06 (5)
Ser266					0.12 (1)	0.01 (1)
Val267	0.07 (3)	0.08 (1)	0.28 (3)	0.66 (4)		0.14 (11)
Glu268	0.02 (1)	0.08 (1)				0.03 (2)
Asn270	0.16 (7)	0.25 (3)	0.37 (4)	0.33 (2)	0.49 (4)	0.25 (20)
Asn355	0.07 (3)		0.28 (3)	0.16 (1)	0.12 (1)	0.10 (8)
Trp358		0.08 (1)			0.12 (1)	0.03 (2)
Trp375						
Pro377		0.08 (1)	0.09 (1)		0.12 (1)	0.04 (3)
Asn378			0.37 (4)		0.12 (1)	0.06 (5)
Ser388	0.26 (11)	0.34 (4)	0.09 (1)			0.20 (16)
Ile389	0.23 (10)	1.17 (14)	4.19 (45)	3.79 (23)	3.18 (26)	1.48 (118)
**Asn449**	0.14 (6)	0.25 (3)	0.19 (2)	1.15 (7)	2.44 (20)	0.48 (38)
Ser450	0.07 (3)	0.25 (3)	0.28 (3)	0.16 (1)	2.44 (20)	0.15 (12)
**Asp451**	0.26 (11)	1.34 (16)	2.61 (28)	18.78 (114)	3.18 (26)	2.46 (196)
Total	4268	1192	1073	607	818	7958

[a]Data were generated with 7958 pH1N1 NA sequences available from GISAID (www.gisaid.org).

[b]Residues in bold represent those identified to be critical for the binding of NA by mAb CD6.

Supplementary Table 4 Primers used for sequencing and mutagenesis of NA gene

Primers for NA gene cloning and sequencing	Sequence[a]
Ba-NA-1	TATTCGTCTCAGGGAGCAAAAGCAGGAGT
345R	CATGATATGAAAGGTTCTCTTATGACA
830R	GCCAGTGTCTGGGTAACAGGAGCATTCCT
Ba-NA-1413R	ATATGGTCTCGTATTAGTAGAAACAAGGAGTTTTTT
830F	AGGAATGCTCCTGTTACCCAGACACTGGC
Primers for site-directed mutagenesis	
P93S	GGCAATTCCTCTCTCTGCTCTGTTAGTGGATGGGCTATATAC
S95A	TCCTCTCTCTGCCCTGTTGCTGGATGGGCTATATACAG
S95N	TCCTCTCTCTGCCCTGTTAATGGATGGGCTATATACAG
R220K	GACACTATCAAGAGTTGGAAGAACAATATATTGAGACAC
N221K	CTATCAAGAGTTGGAGAAAGAATATATTGAGAACACAAGAG
Q250A	ACCGATGGACCAAGTAATGGAGCGGCCTCATACAAGATCTTC
I263V	CAGAATAGAAAAGGGAAAGGTAGTCAAATCAGTCGAAAG
I263K	CAGAATAGAAAAGGGAAAGAAAGTCAAATCAGTCGAAATG
V264T	GAATAGAAAAGGGAAAGATAACCAAATCAGTCGAAATGAATGCCCC
V267I	ATTAGGGGCATTCATTTCTATTGATTTGACTATCTTTCCC
N270D	GTCAAATCAGTCGAAATGGATGCCCCTAATTATCACTATGAG
W375A	AACGGTTTTGAGATGATTGCGGATCCGAACGGATGGACTGGG
W375G	C GGTTTTGAGATGATTGGGGATCCGAACGGATGGACTGGG
P377A	GAGATATTTGGGATGCGAACGGATGGACTGGGACAGAC
N378A	GAGATGATTTGGGATCCGGCCGGATGGACTGGGACAGAC
S388A	GGGACAGACAATAACTTCGCAATAAAGCAAGATATCGTAGG
I389V	GGGACAGACAATAACTTCTCAGTAAAGCAAGATATCGTAGG
N449D	ATATCCTTTTGTGGTGTAGACAGTGACACTGTGGGTTGGTC
N449E	GCATATCCTTTTGTGGTGTAGAGAGTGACACTGTGGGTTGG
N449Q	GCATATCCTTTTGTGGTGTACAGAGTGACACTGTGGGTTGG
N449K	GCATATCCTTTTGTGGTGTAAAAAGTGACACTGTGGGTTGGTC
D451G	TTTTGTGGTGTAGACAGTGGCACTGTGGGTTGGTCTTGGCC
N449D/D451G	TTTTGTGGTGTAGACAGTGGCACTGTGGGTTGGTCTTGGCC

[a]Blue color highlights the nucleotide changes introduced to make the targeted amino acid mutations.

References

1. Salerno, W.J., Seaver, S.M., Armstrong, B.R. & Radhakrishnan, I. MONSTER: inferring non-covalent interactions in macromolecular structures from atomic coordinate data. *Nucleic Acids Res* 32, W566-8 (2004).
2. Krissinel, E. & Henrick, K. Inference of macromolecular assemblies from crystalline state. *J Mol Biol* 372, 774-97 (2007).
3. McDonald, I.K. & Thornton, J.M. Satisfying hydrogen bonding potential in proteins. *J Mol Biol* 238, 777-93 (1994).
